# Glassy dynamics of nanoparticles in semiflexible ring polymer nanocomposite melts

**DOI:** 10.1038/srep44325

**Published:** 2017-03-14

**Authors:** Xiaolin Zhou, Yangwei Jiang, Zhenyu Deng, Linxi Zhang

**Affiliations:** 1 Department of Physics, Zhejiang University, Hangzhou, 310027, China

## Abstract

By employing molecular dynamics simulations, we explore the dynamics of NPs in semiflexible ring polymer nanocomposite melts. A novel glass transition is observed for NPs in semiflexible ring polymer melts as the bending energy (*K*_*b*_) of ring polymers increases. For NPs in flexible ring polymer melts (*K*_*b*_ = 0), NPs move in the classic diffusive behavior. However, for NPs in semiflexible ring polymer melts with large bending energy, NPs diffuse very slowly and exhibit the glassy state in which the NPs are all irreversibly caged be the neighbouring semiflexible ring polymers. This glass transition occurs well above the classical glass transition temperature at which microscopic mobility is lost, and the topological interactions of semiflexible ring polymers play an important role in this non-classical glass transition. This investigation can help us understand the nature of the glass transition in polymer systems.

Polymer nanocomposites, which involve nanoparticles (NPs) dispersed in polymer melts, have received a lot of attention recently because of their potential in fabricating materials with novel mechanical, flame resistance, thermal, electrical, and photonic properties^1^. In order to fully realize their practical potential, it is important to develop a detailed microscopic understanding of dynamical properties of polymer nanocomposites^2^,^3^. As a consequence there have been many investigations to study the dynamics of NPs in polymer melts to optimize properties and to facilitate their processing^4^,^5^,^6^,^7^,^8^,^9^,^10^. Owing to the fact that there are several length scales involved such as the radius of gyration of the polymer chain, the radius of the nanoparticle, and the correlation length of the polymer, it is rather complicated to investigate the dynamical phenomenon of NPs in polymer melts. The NP diffusion coefficient in the large limit follows the Stokes-Einstein (SE) relation, and the corresponding behavior of small NPs is also described by the SE relationship but with a length-scale-dependent viscosity that is smaller than the macroscopic value^11^,^12^. However, the motion of the intermediate sized NPs is faster than SE behavior owing to hoplike motions through the polymer’s entanglement mesh^13^, and faster diffusion for the intermediate sized NPs is also predicted by a microscopic force-level theory^14^. Meanwhile, the dynamical behaviors of NPs in polymer melts are also studied experimentally. Mukhopadhyay *et al*. studied the diffusion coefficient of gold NPs in poly(butyl methacrylate) melts by tracking their motion within a diffraction-limited focus of a laser and found that the gold NPs diffuse 250 times faster than predicted by the SE relation for longer chains^9^. Archer *et al*. reported interesting dynamical behaviors of NPs grafted with PEG in PMMA melts and found that NPs exhibit a transition from diffusive to hyperdiffusive when *M*_*w*_ becomes greater than the entanglement molecular weight^15^. Rubinstein *et al*. proposed a NP hopping mechanism for NP diffusion, in which NPs must overcome a hopping free energy barrier to move and the entanglement strands slip around the NPs resulting in localization of the NP in a neighbouring cage^16^. In a word, the dynamical behaviors of NPs in polymer melts appears to be complicated by hopping effects, length-scale-dependent entanglement forces, polymer-NP interactions, NP shape as well as polymer topological constraints.

Ring polymers are formed by the simple operation of joining together the free ends of a polymer chain, and topological properties manifest themselves on a variety of properties of ring polymers because topological constraints of ring polymer chains decrease the conformational degrees of freedom^17^,^18^,^19^,^20^,^21^,^22^,^23^,^24^,^25^,^26^,^27^. For example, ring polymer melts exhibit self-similar dynamics, yielding a power-law stress relaxation, instead of the entanglement plateau followed by exponential decay observed in entangled linear chains^22^. The rings relax stress much faster than linear polymers and the zero-shear viscosity is found to vary as η_0_~N^1.4^ which is much weaker than the N^3.4^ behavior of linear chains^23^. Moreover, topological interactions of threading can lead to a novel glass transition for the concentrated melts of long flexible ring polymers^24^. The effective interactions exerted on semiflexible ring polymers may lead to interpenetration with increasing concentration^25^, whereas flexible ring polymers adopt crumpled globular conformations in the melt state^26^. Moreover, a novel stack formation with a tube-like structure of quasi-parallel ring is found in semiflexible ring polymer melts^27^. Therefore, polymer chain topological constraints of ring polymers affects the statistical and dynamical properties of polymers seriously, and in this article, we investigate the dynamical behavior of NPs in semiflexible ring polymer nanocomposite melts. Our aim is to study the effects of polymer topological property and polymer stiffness on the dynamical properties of NPs in polymer nanocomposite melts. Fortunately, a novel glassy dynamics of NPs is revealed in the presence of semiflexible non-concatenated, unknotted ring polymer melts.

## Model

In our simulation, a standard bead-spring model is used to model ring polymer chains^28^, and each ring polymer chain consists of N = 30 monomers with a monomer diameter of *σ* and a mass of *m*. To prevent overlap between monomers, a shifted and cut-off Lennard-Jones potential is used for all polymer monomers,


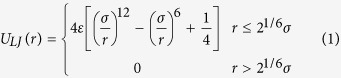


where *r* is the distance between two monomecrs, and *ε* = k_B_T.

All neighbouring monomers are connected by the well-known finitely extensible nonlinear elastic (FENE) potential^29^:


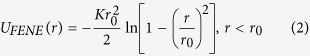


where *r* is the distance between two neighbouring monomers. The parameters *K* and *r*_*0*_ are chosen as *K* = 30k_B_T/σ^2^ and *r*_0_ = 1.5σ^29^.

Angle bending potential between adjacent bonds is used to describe the stiffness of ring polymer chains,


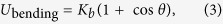


where *θ* is the angle between two neighbouring bonds and the stiffness of ring polymer chains is controlled by varying *K*_*b*_.

The NP/NP and NP/polymer are represented by truncated and shifted Lennard-Jones (LJ) potentials of the form^30^,^31^,^32^,^33^


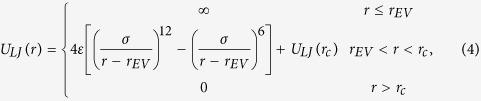


NPs are modeled as spheres of diameter *d* = 5σ, which is widely used in studying the statistical and dynamical properties of polymer nanocomposite melts^30^,^34^,^35^,^36^,^37^, and the density of NPs is same as the ring polymers. The purely repulsive interactions of Weeks-Chandler-Anderson (WCA)^32^ are given by *ε* = 1.0*k*_*B*_*T* and *r*_c_ = 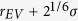 with *r*_*EV*_ = 4σ for NP/NP interactions and *r*_*EV*_ = 2σ for NP/polymer interactions. In order to avoid the competition between the NP/polymer interactions and the topological interactions of semiflexible ring polymers, the purely repulsive interactions for NP/polymer interactions are adopted here. Meanwhile, we only focus on the purely repulsive interactions for NP/polymer interactions here because our aim is to investigate the effects of polymer chain topological constraints of ring polymers on the dynamical properties of NPs in melts. In fact, the purely repulsive interactions for NP/polymer interactions are widely adopted in investigating the statistical properties of polymer nanocomposite melts^30^,^31^,^32^,^33^,^34^,^37^,^38^.

In our simulation, the total number of NPs and polymer monomers are fixed to be N_N_ = 6 and N_P_ = 28800. The NPs and ring polymers are placed randomly in a very large box, and the NPT ensemble was used to compress the system of very low density to the desired equilibrium density. The systems were simulated in the NPT ensemble for long time yielding the desired equilibrium density for different bending energies of polymer chains^30^,^31^,^38^.Then the systems were equilibrated in the NVT ensemble for very long time. Reduced units (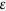 = 1,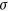 = 1, m = 1, ρ^−3^ = 1, and 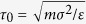 = 1 are chosen to be the units of energy, length, mass, monomer density, and time, respectively) are used and the time step is *τ* = 0.001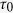. The reduced temperature is T* = 1.0 in units of ε/k_B_ by using a Nose-Hoover thermostat. Periodic boundary conditions were applied during the whole process, and NP coordinates were recorded to calculate some related parameters. All simulations were performed by the open source LAMMPS molecular dynamics package^39^.

## Results and Discussion

**Figure 1:**
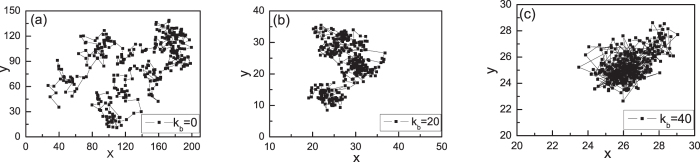
The trajectories of one NP in the projective xy-plane with different bending energies of *K*_*b*_ = 0(**a**), 20 (**b**), and 40 (**c**) in the interval of 10^5^*τ*. Here the monomer number density is *C* = 0.4, and the total number of trajectories is 200.

**Figure 2:**
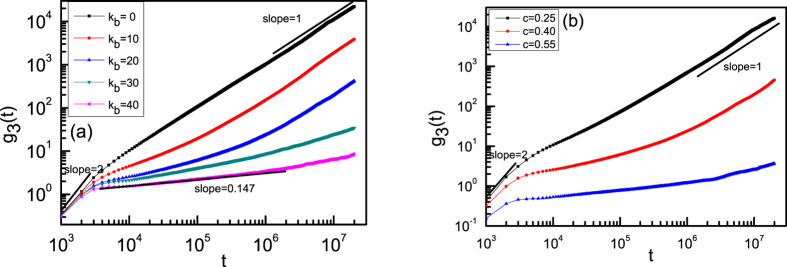
g_3_(t) of NPs in ring polymer melts with different bending energies for *C* = 0.4 (**a**) and with different monomer densities of ring polymers for *K*_*b*_ = 20 (**b**).

Figure 1 shows different trajectories of one NP in the projective xy-plane with different bending energies. NPs are caged in the small region for *K*_*b*_ = 40, see Fig. 1(c). The monomer number density C is defined as C = (N * N_p_)/L^3^, here L is the box size after the systems have equilibrated in the NVT ensemble for very long time^24^. However, NPs can diffuse quickly in flexible ring polymer melts (*K*_*b*_ = 0), see Fig. 1(a). Therefore, the bending energy of ring polymers can seriously affect the diffusion of NPs in ring polymer nanocomposite melts. In fact, the motion of NPs in ring polymer nanocomposite melts can be characterized by the mean-square displacement of the center-of-mass, *g*_3_(*t*), which is defined as


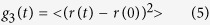


where *r(t)* is the position of a NP at time *t*, *r(0)* is its initial position, and the brackets <  > represent ensemble averaging over many conformations and all NPs. For the case of NPs in flexible ring melts (*K*_*b*_ = 0) with a monomer density of *C* = 0.4, see Fig. 2(a), the motion at short times is ballistic, and *g*_3_(*t*) ~ 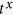 where *x* is close to 2. At later times, the NPs move diffusively and the slope of *g*_3_(*t*) is close to 1. This shows that the NPs move randomly in flexible ring melts with *C* = 0.4. Meanwhile, the similar behavior is also observed for NPs in semiflexible ring melts with a low monomer density of *C* = 0.25, see Fig. 2(b). In fact, for NPs with various bending energies of ring polymer melts at *C* = 0.4 or with various densities of semiflexible ring melts at *K*_*b*_ = 20, the motion at early times is superdiffusive with *x* ≈ 2. For NPs in ring melts with *K*_*b*_ ≤ 20 the motion becomes diffusive at long times with *g*_3_(*t*) ~ *t.* However, for NPs in semiflexible ring melts with large bending energy such as K_b_ ≥ 30, see Fig. 2(a), the NPs are trapped by their neighbouring semiflexible ring polymers, and a plateau emerges at intermediate times where the motion is subdiffusive with 0.147 <*x* < 1. This plateau becomes obvious for *K*_*b*_ = 40 and represents the slowing down of NP motion due to the cage effect of semiflexible ring polymers. This behavior is reminiscent of transient caging motion of particles in a uniformly heated granular fluid^40^, or in a quasi-2D system of bidispersed particles fluidized by a uniform upflow^41^. A Similar dependence of *g*_3_(*t*) has been also observed in trapping motion of NPs in polymer naocomposites due to emerging entanglement constraints^7^. Meanwhile, trapping motion of NPs in nanocomposite melts is also observed for the higher monomer density of *C* = 0.55 with the lower bending energy of *K*_*b*_ = 20, see Fig. 2(b).

Diffusion coefficients are estimated from


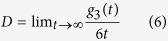


**Figure 3:**
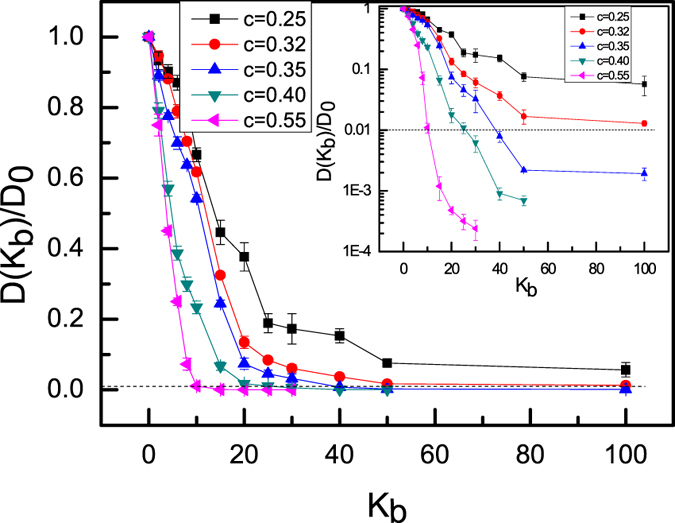
The ratio of *D*(K_b_)/*D*_0_ as a function of *K*_b_ for NPs in ring polymer nanocomposite melts with five monomer densities of *C* = 0.25, 0.32, 0.35, 0.40 and 0.55.

Whose average is taken over all NPs and vanishes when all the NPs are caged by the topological interactions of semiflexible ring polymers^24^. Diffusion coefficients depend on the stiffness of ring polymers seriously, and Fig. 3 shows that the ratio of *D(K*_*b*_*)/D*_*0*_ decreases dramatically with the increase of stiffness of ring polymers (*K*_*b*_). Here *D(K*_*b*_) is the diffusion coefficient of NPs in polymer nanocomposite melts with a stiffness of ring polymers *K*_*b*_. For all systems with high monomer density there exists a critical frozen stiffness (*K*_b_^*^), for which all NPs are permanently trapped by the semiflexible ring polymers, i.e., for semiflexible ring polymer chains with *K*_b_ > *K*_b_^*^. NPs exhibit a transition from (at least partially) liquid, or diffusive, behavior to a glassy state in which the NPs are all irreversibly caged, although free to re-arrange their conformations to some extent. Therefore, it is natural to identify *K*_b_^*^ as the value of *K*_*b*_ at which *D(K*_*b*_) = 0. In practice, we define as “caged” states that display a *D(K*_*b*_) one hundred times smaller than the diffusion coefficient of the flexible ring polymer case, *D*_*0*_. In fact, this method is also applied to investigate the glass transition of long flexible ring polymer melts^24^. From Fig. 3 one can notice that the decay of the D(K_b_) becomes increasingly steeper as the density increases. Meanwhile, the dynamical properties of NPs in polymer nanocomposite melts also rely on the chain length of semiflexible ring polymer chains, and some detailed discussions are given in Supplementary Information. In fact, the ratio of the polymer size to the NP radius plays an important role in the dynamical process of NPs in polymer nanocomposite melts. Of course, the size of semiflexible ring chains depends on the bending energy K_b_. For example, the root-mean-square radius of gyration R_g_ = 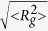 is 2.29 for 30-monomer flexible ring chains (K_b_ = 0) and 4.52 for 30-monomer semiflexible ring chains (K_b_ = 40) with a monomer number density of C = 0.4. Therefore, the ratio of the polymer size to the NP radius η = R_g_/(d/2) = 2R_g_/d = (2 * 2.29)/5 = 0.92 for K_b_ = 0 and η = 1.81 for K_b_ = 40, which is consistent with the experimental result^5^. In the experimental study on dynamics of gold NPs in a polymer melt, the size of gold NP is about 5 nm, and the poly(n-butyl methacrylate) PBMA size is in the range of 2~18 nm, therefore, the ratio η ranges from 0.5 to 3.6^5^, which is close to our simulation results.

**Figure 4:**
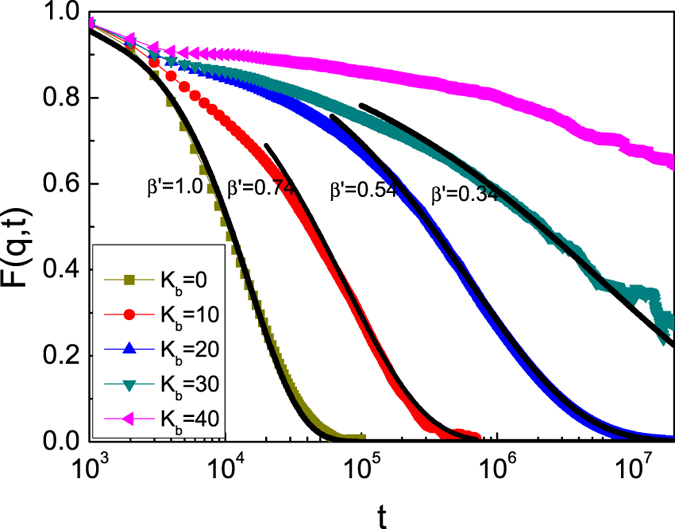
Time dependence of the intermediate scattering function *F*(q, t) for different bending energy of ring polymers with a monomer density of *C* = 0.40. The solid lines are fits to Eqn (8) with different value of β′.

**Figure 5:**
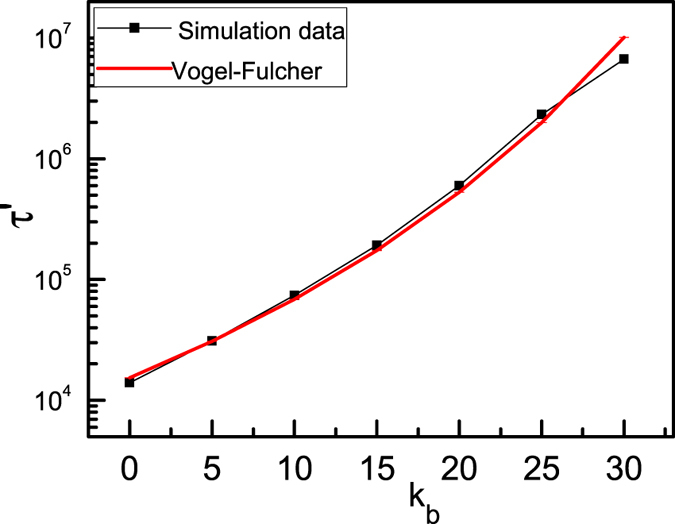
Relaxation time τ′ as a function of K_b_ for a monomer density of semiflexible ring polymers *C* = 0.4.

Incoherent intermediate scattering function is used to study the dynamical process of the system, which is defined as^39^,^42^,^43^,


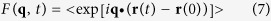


where 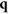 is a wavenumber and the brackets <> are averaged over many conformations and all NPs. In fact, *F*(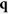, t) is the space-Fourier transforms of the van Hove correlation function^40^, and is widely used in computer simulation of colloid science since it is readily available through light scattering experiments^42^,^43^,^44^,^45^. Figure 4 shows *F*(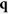, t) for different chain stiffness *K*_*b*_ with *q* = 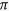/5. For flexible ring polymers, *F*(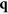, t) decays rapidly as NPs can move diffusively, and NPs are in the liquid phase. For NPs in the glassy phase (*K*_*b*_ 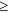 30), *F*(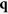, t) decays slowly and it is little decorrelation since NPs are trapped by semiflexible ring polymers. In fact, Fig. 4 shows that *F*(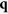,t) includes a two-step relaxation in the intermediate value of *K*_b_: the fast (early time) β relaxation which is the diffusion inside the “cage” and the α relaxation which corresponds to the time it takes for the NP to diffuse out of the “cage”. When *K*_b_ increases, there exists a plateau and the α relaxation occurs at increasing longer timescales. The α relaxation at late times can be described by a Kohlrausch-Williams-Watts (KWW) function^45^


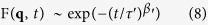


where τ′ is the relaxation time and β′ is the stretching parameter. The relaxation time τ′ is defined as the time it takes for the values of *F*(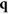. t) to fall to a level of 1/e, i.e., *F*(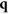,τ′) = 1/e F(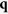.0)^40^. The exponential β′ and the fits of this stretched exponential form to the simulation data are shown as solid lines in Fig. 4. The exponential β′ decreases as the bending energy *K*_b_ increases. For flexible ring polymer melts, as the exponential β′ is close to 1, *F*(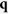. t) can be expressed approximately as exp(−t/τ′), and this is the result for classical diffusion of NPs in liquid phase^46^,^47^, For semiflexible ring polymer melts, the relaxation time τ′ increases abruptly as *K*_b_ increases, and a significant slowing down of the dynamics is observed for large bending energy. Meanwhile, the relaxation time can also be described approximately by the Vogel-Fulcher law^48^,^49^, which is always widely used in studying the glass phase of particles,


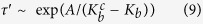


where *A* = 719 and 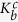 = 74.57 are the fitting parameters. Figure 5 shows that the relaxation times of NPs in nanocomposite melts depend mainly on the stiffness of ring polymers. As NPs go through the transition from the fluid phase to the glassy phase gradually, relaxation time shows no particle feature. However, we make sure that NPs are in the glassy phase for semiflexible ring polymers with large bending energy owing to the caging motion of NPs in semiflexible ring polymer melts

To study the non-Gaussian behavior of the NP diffusion, we compute the following non-Gaussian parameter^50^,


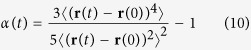


**Figure 6:**
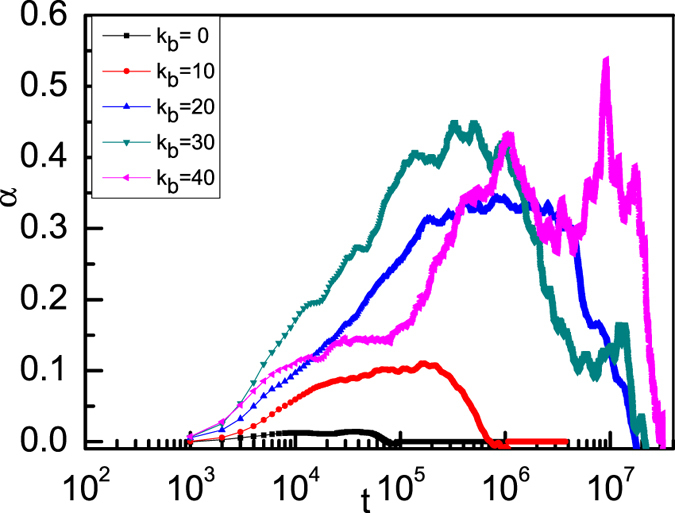
Time-dependent non-Gaussian parameter α for a monomer density of ring polymers *C* = 0.4 with different bending energies.

<….> denotes an ensemble average over all NPs and initial time. This parameter quantifies the deviation from the Gaussian behavior of the probability density function for single-NP diffusion. For flexible ring polymers, the NPs are freely diffusing as the trapping cage isn’t formed by the surrounding ring polymer chains, and α(t) is very small with a typical normal liquid for NPs, see Fig. 6. For semiflexible ring polymers, the motion of each NP is hampered by its ring polymers and becomes subdiffusive, hence, α(t) is nonvanishing, indicating the dynamical heterogeneities. Additionally, we find that the peak height of α(t) increases upon increasing the bending energy *K*_b_. In fact, non-Gaussian dynamics due to cage-escape processes have also been observed in single-particle diffusion in cluster crystals^51^,^52^, glasses^53^,^54^, and in a uniformly heat granular fluid^40^.

Extending an idea originally proposed for spin glasses^55^, one can construct a time-dependent ‘order parameter’ that compares the liquid configuration at two different times^56^,^57^:


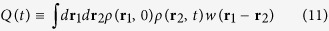


**Figure 7:**
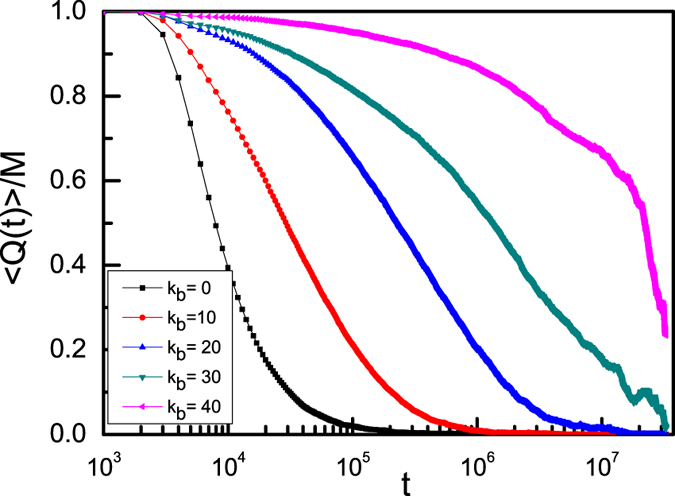
Average time-dependent overlap “order parameter” < Q(t) > /M for a monomer density of ring polymers C = 0.4 with different bending energies.

Here, ***r***_***i***_ in the second equality refers to the position of NP *i*, and 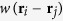 is an “overlap” function which is unity for 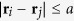, where *a* is associated with the typical vibrational amplitude of the NPs, and we take *a* = 2.5σ^56^,^57^. In fact, *Q*(t) is the number of “overlaping” NPs when configurations of the system at *t* = 0 and at a later time *t* are compared, and *Q(t)* counts the number of NPs that either remain within a distance *a* of their original position, or are replaced by another NP in an interval *t*. Figure 7 shows that for NPs in semiflexible ring polymer melts, *Q*(t) is characterized by a two-step relaxation, commonly observed in the intermediate scattering function *F*(q, t) in Fig. 4, as a result of the transient caging of NPs. At short times, NPs oscillate in a region smaller than the overlap radius a, i.e., < *Q(t)* > /*M* is close to 1, especially for large bending energy. Here M is the number of NPs. Meanwhile, there exists a short, initial relaxation of < Q(t) > and a longer, secondary relaxation. Therefore, we can conclude that the NPs in the semiflexible ring melts are caged and the liquid-glass phase transition occurs when the bending energy *K*_b_ increases. In general, the glass transition is associated with a reduction in the temperature of liquids or by an increase in density of granular materials. Therefore, there exist a critical temperature (i.e.,T_g_) or a critical density (C*) of granular material for the classical glass transition. However, in this work, we obverse another type of glass transition for NPs in semiflxible ring melts and the glass transition occurs *only* with increasing the bending energy of semiflxible ring polymers. Because that this glass transition takes place well above the classical glass transition temperature at which microscopic mobility is lost, we can choose the system temperature to be fixed at T* = 1.0. In fact, a topologically driven glass in long flexible ring polymers has been observed by Michieletto and Turner^24^, and they found that a concentrated solution of long ring polymers can be driven to a kinetically arrested state by randomly pinning a small fraction of ring polymers. In their model, the system temperature is also fixed, and the glass transition can’t be determined^24^, which is different from the classical glass transition.

**Figure 8:**
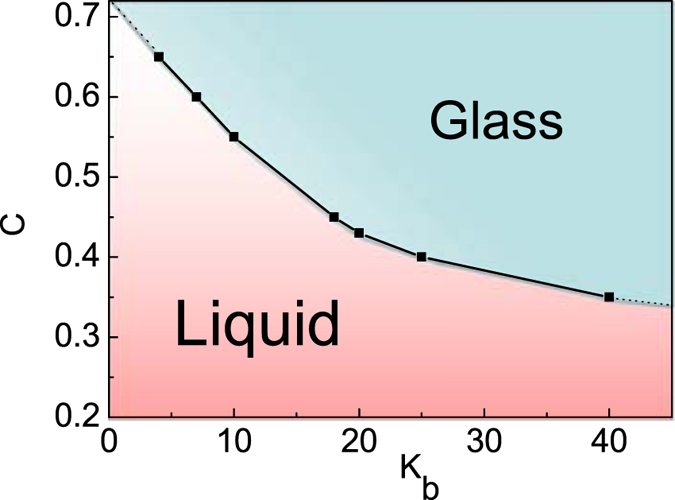
Phase diagram of NPs in semiflexible ring polymer nanocomposite melts relies on monomer density (*C*) and bending energy (*K*_b_) of semiflexible ring polymers.

The behaviors of diffusion coefficient in Fig. 3 show an increasing steeper dependence on the monomer density of ring polymers as the chain stiffness of ring polymers *K*_b_ increases. This implies that systems made of semiflexible ring polymer nanocomposite with large bending energy become extremely sensitive to the monomer density of ring polymers. Figure 8 shows that the phase diagram of NPs in semiflexible ring polymer melts depends on the bending energy (*K*_b_) as well as the monomer density (*C*) of ring polymers. There exists a critical density of polymer chains in nanocomposite melts: which is located at *C*^min^ ≈ 0.33, and is estimated according to the ratio of *D(K*_*b*_*)/D*_*0*_ in Fig. 3. For C = 0.35, the glass transition occurs at K_b_ = 40, while for C = 0.32, the ratio of *D(K*_*b*_*)/D*_*0*_ is 0.0129 at K_b_ = 100, which is greater than 0.01 yet. Therefore, we estimate that the absolute minimum *C*^min^ is approximately equal to 0.33, which is an extrapolation of the data from K_b_ = 40. This is the minimal monomer density of ring polymer chains to form the glass phase for NPs in ring polymer nanocomposite melts. In the glass phase, the NPs are caged by the topological interactions of semiflexible ring polymers^22^ and this is a novel phase transition because this occurs well above the classical glass transition temperature at which microscopic mobility is lost^24^. In fact, the topological interactions of semiflexible ring polymers play an important role in this non-classical glass transition. The stack structures for semiflexible ring polymers in nanocomposite melts hinder the motion of NPs^27^, and this is the main reason to form “caging” motion for NPs in semiflexible ring polymer melts.

## Concluding Remarks

Dynamical behaviors of NPs immersed in semiflexible ring polymer melts with various bending energies are investigated by using molecular dynamics simulations. A novel glass transition for NPs in semiflexible ring polymer melts is observed as the bending energy (*K*_b_) increases. For NPs in flexible ring polymers, the classical dynamics occurs and the motion of NPs is diffusive. However, for NPs in semiflexible ring polymer melts with larger bending energy, the NPs exhibit a transition from liquid behavior to a glassy state in which the NPs are all irreversibly caged. Moreover, the relaxation times of NPs in semiflexible ring polymer melts can be expressed approximately as 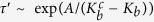, which is similar with the Vogel-Fulcher law. Meanwhile, the glass transition for NPs in semiflexible ring nanocomposite melts also relies on the monomer density of semiflexible ring polymers in melts. There exists a critical monomer density *C*^min^ with *C*^min^ ≈ 0.33, which is the minimal density of ring polymer chains to form the glass phase for NPs in ring polymer nanocomposite melts. As our glass transition occurs well above the classical glass transition temperature at which microscopic mobility is lost, our glass transition is different from classical glass transition, and there doesn’t exist the glass transition temperature for our simulation system. This investigation can help understand the nature of the glass transition in polymeric systems.

## Additional Information

**How to cite this article**: Zhou, X. *et al*. Glassy dynamics of nanoparticles in semiflexible ring polymer nanocomposite melts. *Sci. Rep.* **7**, 44325; doi: 10.1038/srep44325 (2017).

**Publisher's note:** Springer Nature remains neutral with regard to jurisdictional claims in published maps and institutional affiliations.

## Supplementary Material

Supplementary Information

## References

[b1] Balazs A. C., T. Emrick T. & Russell T. P. Nanoparticle polymer composites: where two small worlds meet. Science 314, 1107 (2006). 17110567 10.1126/science.1130557

[b2] Liu J., Zhang L., Cao D. & Wang W. Static, rheological and mechanical properties of polymer nanocomposites studied by computer modeling and simulation. Phys. Chem. Chem. Phys. 11, 11365 (2009). 20024405 10.1039/b913511a

[b3] Egorov S. A. Anomalous nanoparticle diffusion in polymer solutions and melts: a mode-coupling theory study. J. Chem. Phys. 134, 084903 (2011). 21361555 10.1063/1.3556749

[b4] Omari R. A., Abeese R. A., Grabowski A. M., & Mukhopadhyay A. Diffusion of nanoparticles in semidilute and entangled polymer solutions. J. Phys. Chem. B 113, 8449 (2009). 19476342 10.1021/jp9035088

[b5] Grabowski C. A., Adhikary B. & Mukhopadhyay A. Dynamics of gold nanoparticles in a polymer melt. Appl. Phys. Lett. 94, 021903 (2009).

[b6] Wyart F. B. & de Gennes P. G. Viscosity at small scales in polymer melts. Eur. Phys. J. E 1, 93 (2000).

[b7] Kalathi J. T., Yamamoto U., Schweizer K. S., Grest G. S. & Kumar S. K. Nanoparticle diffusion in polymer nanocomposites. Phys. Rev. Lett. 112, 108301 (2014). 24679329 10.1103/PhysRevLett.112.108301

[b8] Kalathi J. T., Kumar S. K., Rubinstein M. & Grest G. S. Rouse mode analysis of chain relaxation in polymer nanocomposites. Soft Matter 11, 4123 (2015). 25939276 10.1039/c5sm00754b PMC4840937

[b9] Grabowski C. A. & Mukhopadhyay A. Size effect of nanoparticle diffusion in a polymer melt. Macromolecules 47, 7238 (2014).

[b10] Jee A. Y., Curtis-Fisk J. L. & Granick S. Nanoparticle diffusion in methycellulose thermoreversible association polymer. Macromolecules 47, 5793 (2014).

[b11] Tuteja A., Mackay M. E., Narayanan S., Asokan S. & Wong M. S. Breakdown of the continuum Stokes-Einstein relation for nanoparticle diffusion. Nano. Lett. 7, 1276 (2007). 17397233 10.1021/nl070192x

[b12] Ziebacz N., Wieczorek S. A., Kalwarczyk T., Fiakowski M. & Holyst R. Crossover regime for the diffusion of nanoparticles in polyethylene glycol solutions: influence of the depletion layer. Soft Matter 7, 7181 (2011).

[b13] Cai L. H., Panyukov S. & Rubinstein M. Mobility of nonsticky nanoparticles in polymer liquids. Macromolecules 44, 7853 (2011). 22058573 10.1021/ma201583q PMC3205984

[b14] Yamamoto U. & Schweizer K. S. Theory of nanoparticle diffusion in unentangled and entangled polymer melts. J. Chem. Phys. 135, 224902 (2011). 22168722 10.1063/1.3664863

[b15] Mangal R., Srivastava S., Narayana S. & Archer L. Size-dependent particle dynamics in entangled polymer nanocomposites. Langmuir 32, 596 (2016). 26694953 10.1021/acs.langmuir.5b03311

[b16] Cai L. H., Panyukov S. & Rubinstein M. Hopping diffusion of nanoparticles in polymer matrices. Macromolecules 48, 847 (2015). 25691803 10.1021/ma501608x PMC4325603

[b17] Pioer P., Bacova P., Moreno A. J., Likos C. N. & Blaak R. Anisotropic effective interactions and stack formation in mixtures of semiflexible ring polymers. Soft Matter 12, 4805 (2016). 27117080 10.1039/c6sm00430j

[b18] Pioper P., Likos C. N., Moreno A. J. & Blaak R. An anisotropic Effective Model for the Simulation of Semiflexible Ring Polymers. Macromolecules 48, 4983 (2015). 26240439 10.1021/acs.macromol.5b00603 PMC4519991

[b19] Narros A., Likos C. N., Moreno A. J. & Capone B. Multi-blob coarse graining for ring polymer solutions. Soft Matter 10, 9601 (2014). 25356818 10.1039/c4sm01904k

[b20] Slimani M. Z., Bacova P., Bernabei M., Narros A. & Likos C. N. Cluster glasses of semiflexible ring polymers. ACS Macro. Lett. 3, d611 (2014). 10.1021/mz500117v PMC4111402 25083314

[b21] Narros A., Moreno A. J. & Likos C. N. Effects of knots on ring polymers in solvents of varying quality. Macromolecules 46, 3654 (2013). 23729865 10.1021/ma400308x PMC3667624

[b22] Kapnistos M., Lang M., Vlassopoulos D., Pyckhout-Hintzen W., Richter D., Cho D., Chang T. & Rubinstein M. Unexpected power-law stress relaxation of entangled ring polymers. Nat. Mater. 7, 997 (2008). 18953345 10.1038/nmat2292 PMC4819970

[b23] Halverson J. D., Lee W. B., Grest G. S., Grosberg A. Y. & Kremer K. Molecular dynamics simulation study of nonconcatenated ring polymers in a melt. II. Dynamics. J. Chem. Phys. 134, 204905 (2011). 21639475 10.1063/1.3587138

[b24] Michieletto D. & Turner M. S. A topologically driven glass in ring polymers. Proc. Natl Acad Sci USA 113, 5195 (2016). 27118847 10.1073/pnas.1520665113 PMC4868430

[b25] Bernabei M., Bacova P. & Moreno A. J. Fluids of semiflexible ring polymers: effective potentials and clustering. Soft Matter 9, 1287 (2013).

[b26] Reigh S. Y. & Yoon D. Y. Single-molecule Imaging reveals topology dependent mutual relaxation of polymer chains. ACS Macro Lett. 2, 296 (2013). 10.1021/mz300587v 35581754

[b27] Poier P., Bačová P. & Moreno A. J. Anisotropic effective interactions and stack formation in mixtures of semiflexible ring polymers. Soft Matter. 12, 4805 (2016). 27117080 10.1039/c6sm00430j

[b28] Kremer K. & Grest G. S. Dynamics of entangled linear polymer melts: A molecular‐dynamics simulation. J. Chem. Phys. 92, 5057 (1990).

[b29] Grest G. S. & Kremer K. Molecular dynamics simulation for polymers in the presence of a heat bath. Phys. Rev. A 33, 3628 (1986). 10.1103/physreva.33.3628 9897103

[b30] Smith J. S., Bedrov D. & Smith G. D. *Compos.* A molecular dynamics simulation study of nanoparticle interactions in a model polymer-nanoparticle composite. Composites Sci. Technol. 63, 1599 (2003).

[b31] Liu J., Cao D. & Zhang L. Molecular dynamics study on nanoparticle diffusion in polymer melts: a test of the Stokes-Einstein Law. J. Phys. Chem. C, 112, 6653 (2008)

[b32] Weeks J. D., Chandler D. & Andersen H. C.Role of repulsive forces in determining the equilibrium structure of simple liquids. J. Chem. Phys. 54, 5237 (1971).

[b33] Jiang Y., Zhang D., He L. & Zhang L. X. Entropic Interactions in semiflexible polymer nanocomposite melts. J. Phys. Chem. B 120, 572 (2016). 26720713 10.1021/acs.jpcb.5b09551

[b34] Bedrov D., Smith G. D. & Smith J. S. Matrix-induced nanoparticle interactions in a polymer melt: A molecular dynamics simulation study. J. Chem. Phys. 119, 10438 (2003).

[b35] Feng Y. C., Zou H., Tian M., Zhang L. Q. & Mi J. G. Relation between dispersion and conductivity of polymer nanocomposites: A molecular dynamics study. J. Phys. Chem. B 116, 13081 (2012). 23057420 10.1021/jp305815r

[b36] Liu J., Gao Y. Y., Cao D. P. & Guo Z. H. Nanoparticle dispersion and aggregation in polymer nanocomposites: Insight from molecular dynamics simulation, Langmuir 27, 7926 (2011). 21595451 10.1021/la201073m

[b37] Shendruk T. N., Bertrand M., Harden J. L., Slater G. W. & de Haan H. W. Coarse-grained molecular dynamics simulations of depletion-induced interactions for soft matter system, J. Chem. Phys. 141, 244910 (2014). 25554183 10.1063/1.4903992

[b38] Liu J., Cao D., Zhang L. & Wang W. Time−temperature and time-concentration superposition of nanofilled elastomers: A molecular dynamics study. Macromolecules 42, 2842 (2009).

[b39] Plimpton S. Fast parallel algorithms for short-range molecular dynamics. J. Comput. Phys. 117, 1 (1995).

[b40] Reis P. W., Ingale R. A. & Shattuck M. D. Caging dynamics in a granular fluid. Phys. Rev. Lett. 98, 188301 (2007). 17501613 10.1103/PhysRevLett.98.188301

[b41] Abate A. R. & Durian, D. J. Approach to jamming in an air-fluidized granular bed. Phys. Rev. E 74, 031308 (2006). 10.1103/PhysRevE.74.031308 17025624

[b42] Pusey P. N. & van Megen W. Dynamic light scattering by non-ergodic media. Physica A 157, 705 (1989).

[b43] van Megen W. & Underwood S. M. Dynamic-light-scattering study of glasses of hard colloidal spheres. Phys. Rev. E 47, 248 (1993). 10.1103/physreve.47.248 9959998

[b44] Hansen J. P. & McDonald I. R. Theory of Simple Liquids, Academic, London (1986).

[b45] Horbach J. & Kob W. Relaxation dynamics of a viscous silica melt: The intermediate scattering functions. Phys. Rev. E 64, 041503 (2001). 10.1103/PhysRevE.64.041503 11690029

[b46] Marty G. & Daushot O. Subdiffusion and cage effect in a sheared granular material. Phys. Rev. Lett. 94, 015701(2005). 15698097 10.1103/PhysRevLett.94.015701

[b47] Daushot O., Marty G. & Biroli G. Dynamical heterogeneity close to the jamming transition in a sheared granular material. Phys. Rev. Lett. 95, 265701 (2005). 16486371 10.1103/PhysRevLett.95.265701

[b48] Vogel H. The law of the relation between the viscosity of liquids and the temperature. Z. Phys. 22, 645 (1921).

[b49] Fulcher G. S. Analysis of recent measurements of the viscosity of glasses. J. Am. Ceram. Soc. 6, 339 (1925).

[b50] Rahman A. Correlations in the motion of atoms in liquid argon. Phys. Rev. 136, A405 (1964).

[b51] Moreno A. J. & Likos C. N. Diffusion and relaxation dynamics in cluster crystals. Phys. Rev. Lett. 99, 107801 (2007). 17930408 10.1103/PhysRevLett.99.107801

[b52] Matena R., Dijkstra M. & Patti A. Non-Gaussian dynamics in smectic liquid crystals of parallel hard rods. Phys. Rev. E 81, 021704 (2010). 10.1103/PhysRevE.81.021704 20365579

[b53] Weeks E. R. & Weitz D. A., Properties of cage rearrangements observed near the colloidal glass transition. Phys. Rev. Lett. 89, 095704 (2002). 12190415 10.1103/PhysRevLett.89.095704

[b54] Kegel W. K. & van Blaaderen A. Direct observation of dynamical heterogeneities in colloidal hard-sphere suspensions. Science 287, 290 (2000). 10634780 10.1126/science.287.5451.290

[b55] Franz S. & Parisi G. Phase diagram of coupled glassy systems: A mean-field study. Phys. Rev. Lett. 79, 2486 (1997).

[b56] Lacevic N., Starr F. W., Schroder T. B., Novikov V. N. & Slotzer S. C. Phys. Rev. E 66, 030101 (2002). 10.1103/PhysRevE.66.030101 12366089

[b57] Lacevic N., Starr F. W., Schroder T. B. & Slotzer S. C. Spatially heterogeneous dynamics investigated via a time-dependent four-point density correlation function. J. Chem. Phys. 119, 7372 (2003).

