# Supplementary material for: Glassy dynamics of nanoparticles in semiflexible ring polymer nanocomposite melts
Source: Sci Rep. 2017 Mar 14;7:44325. doi: 10.1038/srep44325 (PMC5349566; doi:10.1038/srep44325)
Supplement: Supplementary Information [file srep44325-s1.doc]

**Supplementary Information**

**Glassy dynamics of nanoparticles in semiflexible ring polymer nanocomposite melts**

Xiaolin Zhou, Yangwei Jiang, Zhenyu Deng, Linxi Zhang^*^

Department of Physics, Zhejiang University, Hangzhou, 310027, China

*Corresponding author. E-mail: lxzhang@zju.edu.cn.

**I. Effects of chain length on the dynamical behaviors of nanoparticles in semiflexible ring polymer nanocomposite melts**

The dynamical behaviors of nanoparticles in semiflexible ring polymer nanocomposite melts with different chain lengths of N=10, 30, and 60 are also investigated and the results of g_3_(t) are shown in Fig. S1. Here the monomer number density is C=0.4, and the diameter of NPs is d=5σ. For short ring chains of N=10, NPs diffuse fast even for large chain stiffness of k_b_=500, see Fig S1-(I). However, for long ring chains (N=60), NPs moves also very slowly for k_b_>10, see Fig. S1-(III). Fig. S2 shows that the glassy dynamics of NPs in nanocomposite melts occurs easily for longer ring polymer chains. In fact, if the ring polymer chains only consist of several monomers, for example, N=1, or 3, this means that the ring polymer chains can be regarded as small NPs and this system only consists of small and large NPs, therefore, the dynamical behaviours of this system don’t depend on the stiffness of ring chains and the glassy dynamics can’t occur because this system can be regarded as granular materials with a low density of C=0.4, whose density is much lower than the critical density for the glass transition of granular materials. However, the glass transition occurs easily for NPs in longer ring polymer melts. Therefore, the chain length of ring polymer chains affects the dynamical behaviours of NPs in nanocomposite melts because the topological interactions of semiflxible ring polymers in melts rely on the chain length of ring chains seriously.^1^

Fig. S1. g_3_(t) of NPs in ring polymer melts with various bending energies for different chain lengths N=10 (I), N=30(II), and N=60(III). Here C=0.4.

Fig. S2. The ratio of D(K_b_)/D_0_ as a function of K_b_ for NPs in semiflexible ring polymer nano- composite melts with different chain lengths N. Here C=0.4.

**II.** **Dynamical behavior of ring polymer chains in nanocomposite melts**

The dynamical behavior of NPs in nanocomposite melts also rely on the dynamical properties of ring polymer chains in nanocomposite melts, and the dynamical process of ring polymer chains are also studied here. Fig. S3 shows that the semiflexible ring chains also diffuse very slow for large bending energy and undergo the glass transition when the bending energy increases due to the topological interactions of semiflexible ring chains.^1-3^ This investigation can help us understand the glassy dynamics of NPs in semiflexible ring polymer nanocomposite melts well.

Fig. S3. g_3_(t) of NPs and ring polymer chains with different bending energies in nanocomposite melts. Here C=0.4.

**References**

1. D. Michieletto, and M. S. Turner, *Proc. Natl. Acad. Sci. U. S. A.* **113**, 5195(2016).

2. W. C. Lo, and M. S. Turner, *EPL*, **102**, 58005(2013).

3. D. Micheletto, D. Marenduzzo, E. Orlandini, G. P. Alexander, and M. S. Turner, *ACS. Macro. Lett.* **3**, 255(2014).
